# Digital PCR Quantification of a Circulating *RBP3* and *CRX* RNA Signature Establishes a Liquid Biopsy Framework for Precision Monitoring of Retinoblastoma

**DOI:** 10.3390/ijms27104177

**Published:** 2026-05-08

**Authors:** Thais Biude Mendes, Indhira Dias Oliveira, Francine Tesser Gamba, Fernanda Teresa Lima, Bruna Fernanda Silva Cardoso Morales, Carla Renata Pacheco Donato Macedo, Luiz Fernando Teixeira, Silvia Regina Caminada de Toledo

**Affiliations:** 1 Genetics Laboratory, Pediatric Oncology Institute (IOP/GRAACC), Federal University of Sao Paulo, Sao Paulo 04039-001, SP, Brazil; 2 National Science and Technology Institute for Children’s Cancer Biology and Pediatric Oncology—INCT BioOncoPed, Porto Alegre 90010-150, RS, Brazil; 3 Department of Gynecology, Federal University of Sao Paulo, Sao Paulo 04024-002, SP, Brazil; 4 Institute of Pediatric Oncology (IOP/GRAACC), Sao Paulo 04023-062, SP, Brazil; 5 Ophthalmology Department, Universidade Federal de São Paulo, Sao Paulo 04023-062, SP, Brazil

**Keywords:** retinoblastoma, liquid biopsy, digital PCR, *CRX*, *RBP3*

## Abstract

Retinoblastoma (RB) is the most common intraocular malignancy of childhood, yet molecular assessment of disease dissemination and minimal residual disease (MRD) remains challenging due to the contraindication of intraocular biopsy. Here, we evaluate the feasibility of cell-free RNA (cfRNA)- and circulating tumor cell RNA (ctcRNA)-based liquid biopsy for the sensitive detection of disseminated retinoblastoma using digital PCR (dPCR) targeting the retina-specific markers *CRX* and *RBP3*. We analyzed 433 bone marrow (BM), peripheral blood (PB) and cerebrospinal fluid (CSF) samples collected longitudinally from 50 patients with RB. dPCR assays demonstrated high analytical sensitivity. cfRNA detection showed complete sensitivity and negative predictive value in bone marrow compared with myelogram analysis, frequently identifying molecular positivity in cytologically negative samples. In cerebrospinal fluid, cfRNA detection was highly specific but less sensitive, reflecting compartment-specific biological constraints. Longitudinal analysis revealed that changes in *CRX* and *RBP3* ctcRNA levels closely tracked treatment response, preceded cytological evidence of bone marrow involvement in several cases, and identified molecular persistence or re-emergence during follow-up, including after hematopoietic stem cell transplantation. Together, these findings demonstrate that cfRNA- and ctcRNA-based liquid biopsy using *CRX* and *RBP3* enables sensitive and dynamic detection of disseminated retinoblastoma, particularly in bone marrow, and supports its potential utility for MRD monitoring. Longitudinal patient analyses will be required to define prognostic thresholds and establish the clinical role of this approach in risk stratification and long-term surveillance.

## 1. Introduction

Retinoblastoma is the most common intraocular malignancy of childhood and arises from retinal precursor cells that undergo malignant transformation during early retinal development [1,2]. Despite substantial improvements in clinical outcomes, molecular interrogation of the disease remains constrained by the inability to obtain tumor tissue, as intraocular biopsy is contraindicated due to the risk of extraocular dissemination [3,4,5,6,7]. There is a growing need for minimally invasive technologies that detect tumor-derived molecular features associated with minimal residual and disseminated disease.

In this context, liquid biopsy has emerged as a promising strategy for the genomic and transcriptomic characterization of solid tumors, enabling the analysis of tumor-derived nucleic acids in body fluids. This approach allows longitudinal disease monitoring and captures the molecular spatial heterogeneity of tumors without the need for direct tissue sampling [8,9]. Molecular profiling through liquid biopsy encompasses the evaluation of circulating tumor cells (CTCs), as well as cell-free DNA (cfDNA) and cell-free RNA (cfRNA) [10].

Cell-free RNA (cfRNA) represents a dynamic class of circulating biomarkers that captures gene expression patterns released from tumor cells through apoptosis, necrosis, or active secretion [11,12]. Unlike genomic alterations, cfRNA profiles may provide real-time information on cellular differentiation states and residual malignant activity [12]. However, the low abundance and fragmented nature of cfRNA necessitate highly sensitive and quantitative analytical platforms.

Digital PCR (dPCR) enables absolute quantification of low-copy-number nucleic acids with high analytical sensitivity and precision, making it particularly suited for the detection of rare tumor-derived transcripts in liquid biopsy samples. When applied to cfRNA analysis, dPCR allows for the reliable measurement of retina-specific gene expression even in the context of minimal residual disease, where tumor burden is expected to be low.

In retinoblastoma, tumor-derived material has been identified in the aqueous humor (AH), peripheral blood (PB) and cerebrospinal fluid (CSF), supporting the feasibility of molecular disease assessment through minimally invasive sampling [13,14,15,16,17]. Although most studies have focused on DNA-based analyses, including *RB1* mutations and somatic copy number alterations, comparatively limited attention has been directed towards circulating RNA species, despite their potential to reflect active transcriptional programs and tumor cell identity [17,18,19,20].

Several studies have demonstrated that gene expression analysis can serve as a sensitive and specific biomarker for assessing disseminated minimal disease (DMD) in RB [21,22,23,24,25]. The *cone–rod homeobox transcription factor* gene (*CRX*) and *retinol*-*binding protein 3* gene (*RBP3*) are highly specific markers of retinal cell identity. *CRX* functions as a master regulator of photoreceptor gene networks, whereas *RBP3* is a key component of the interphotoreceptor matrix [20,22,23,24,25,26,27]. The detection of *CRX* and *RBP3* cfRNA in liquid biopsy specimens may therefore serve as a molecular surrogate for tumor-derived retinal material and provide insight into the persistence of residual malignant cell populations [20,21,22].

In this study, the feasibility of detecting *CRX* and *RBP3* ctcRNA and cell-free RNA in liquid biopsy samples from patients with retinoblastoma using digital PCR was evaluated. Samples were obtained at baseline, before the initiation of chemotherapy, and subsequently after every two chemotherapy cycles. By integrating cfRNA and ctcRNA analysis with dPCR-based quantification, this work aimed to establish a sensitive and minimally invasive molecular framework for the detection of minimal residual disease and the characterization of tumor-derived retinal transcriptional signatures. Overall, cfRNA- and ctcRNA-based liquid biopsy targeting *CRX* and *RBP3* enables sensitive detection of disseminated retinoblastoma.

## 2. Results

### 2.1. Limit of Detection (LoD) of dPCR System

To assess the suitability of this approach for monitoring minimal disseminated disease, we first standardized the assay to determine the limit of detection (LoD) for *RBP3* and *CRX*. A serial dilution (20%, 10%, 1%, 0.1%, 0.01% and 0.001%) of Y79 RNA in a background of KHOS RNA was used to establish fluorescence thresholds of 15,000 and 10,000 FAM units for *RBP3* and *CRX*, respectively (Supplementary Figure S1A). Linear regression analysis showed strong linearity across the dilution range, with an R^2^ of 0.9978 and *p* < 0.0001 for both targets (Supplementary Figure S1B,C).

Based on these thresholds, seven independent experiments identified an LoD of 0.01% for both genes and, using 10 ng of input RNA, 1.23 copies/µL of *RBP3* and 0.60 copies/µL of *CRX*, indicating high sensitivity and specificity of the assay. Further validation was obtained using RNA from BM samples of patients with retinoblastoma and confirmed BM infiltration, in which both genes were consistently detected (Supplementary Figure S2). All experiments were performed in quintuplicate with appropriate positive and negative controls, and target detection was achieved in 100% of replicates, demonstrating robust assay performance and reproducibility (Supplementary Figure S3).

### 2.2. Patient and Sample Characteristics

**Figure 1:**
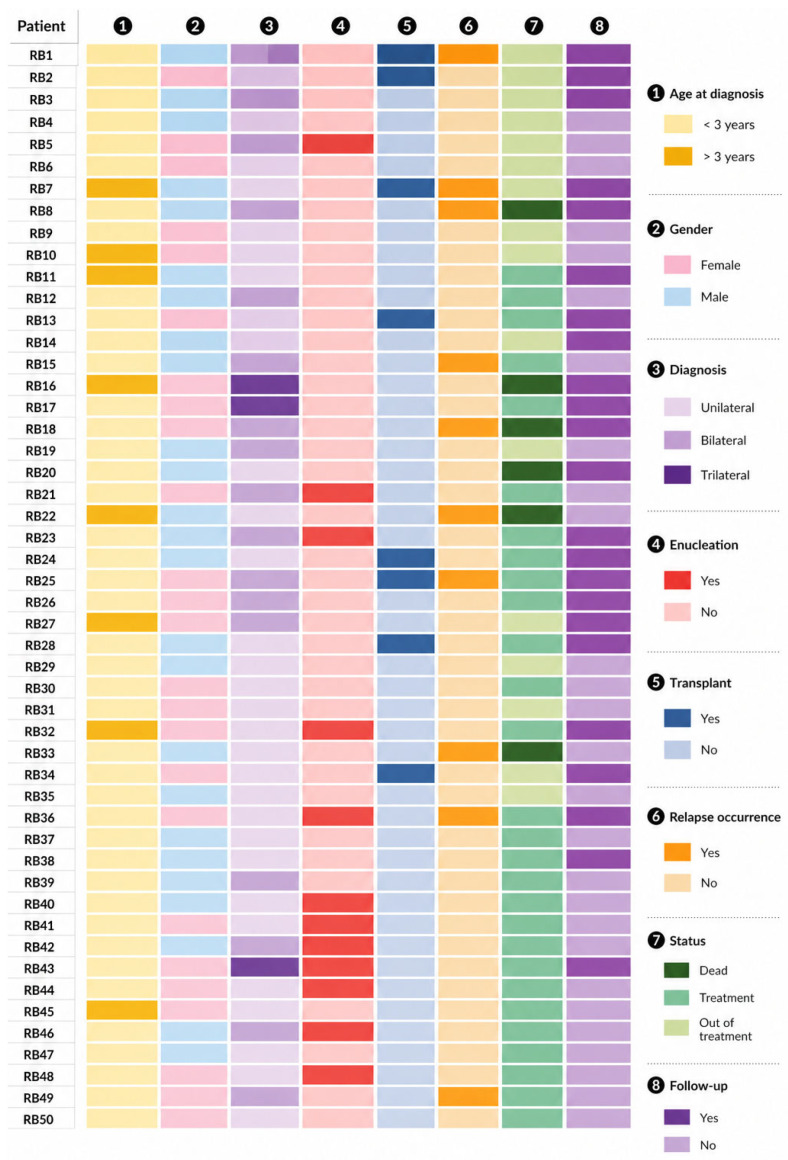
Landscape of 50 RB patient and sample characteristics.

This study included 50 patients diagnosed with retinoblastoma, of whom 50% were female and 50% were male (Figure 1). Two patients were diagnosed in 2017, and 48 patients were diagnosed between January 2024 and December 2025. The median age at diagnosis was 26 months (2.2 years). Of the total cohort, 60% (*n* = 30) presented with unilateral retinoblastoma, 34% (*n* = 17) with bilateral disease, and 6% (*n* = 3) with trilateral retinoblastoma (Table 1).

Among patients with unilateral disease, 63% presented with intraocular tumors and 37% with extraocular involvement. Enucleation was performed in 80% of cases (*n* = 24), whereas 20% (*n* = 6) had not undergone enucleation at the time of analysis. Recurrence occurred in four patients (13%), including one with extensive metastatic infiltration involving craniofacial soft tissues, intraorbital bones, and multiple vertebral bodies. Overall, 20% of the cohort (*n* = 6) underwent hematopoietic stem cell transplantation (HSCT) (Table 1).

Among patients with bilateral retinoblastoma, 53% (*n* = 9) had intraocular disease and 47% (*n* = 8) had extraocular involvement. Enucleation of either eye was not performed in 29% of these patients (*n* = 5). Disease recurrence or metastasis was observed in 35% of bilateral cases (*n* = 6), and two patients died. HSCT was performed in two patients (Table 1). Of the three patients with trilateral retinoblastoma (bilateral retinoblastoma with pineoblastoma), two had extraocular disease and one had intraocular disease; enucleation was performed in two of them, and one patient died during treatment.

A total of 70 eyes from 50 patients were evaluated. Most eyes (70%, *n* = 49) were classified as having Group E retinoblastoma according to the International Intraocular Retinoblastoma Classification (IIRC), including 27 eyes from patients with unilateral disease and 22 from patients with bilateral or trilateral disease. The remaining eyes from bilateral cases were classified as Group A (*n* = 3), Group C (*n* = 5), and Group D (*n* = 7) (Table 1).

A total of 433 samples, including bone marrow (BM), peripheral blood (PB), and cerebrospinal fluid (CSF), were obtained from the 50 patients and analyzed during the course of treatment. These comprised 217 BM samples (109 right and 108 left), 106 PB samples, and 110 CSF samples. Of these, 191 samples (BM, PB, and CSF) were collected at baseline, before treatment initiation, from all 50 patients. Six patients (RB7, RB18, RB25, RB33, RB36 and RB49) were enrolled after disease recurrence, and BM, PB, and CSF samples were collected before initiation of relapse-directed therapy. The remaining 324 samples were collected longitudinally during treatment from 22 patients.

### 2.3. Correlation Between dPCR, Myelogram and CSF Cytology Results

Results obtained by digital PCR (dPCR) were compared with those from conventional methods used to assess minimal disseminated disease, including myelogram analysis for BM samples and oncological cytology for CSF. In total, 433 samples were analyzed, and the overall comparison is summarized in Table S1.

**Figure 2:**
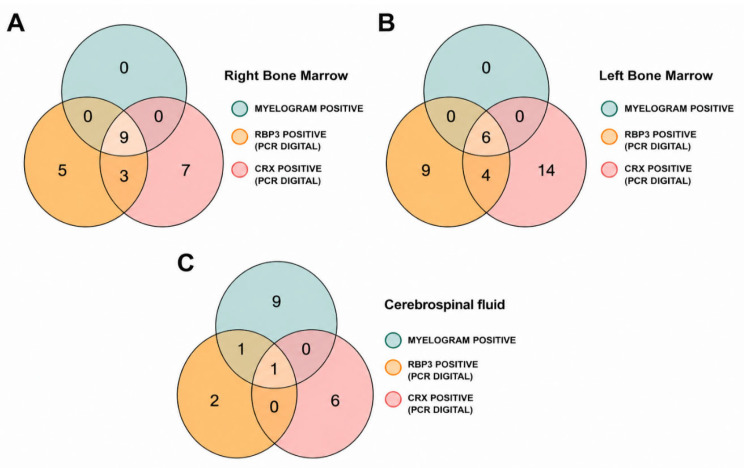
Venn diagram on concordance between myelogram and digital PCR of bone marrow (BM) on the right (**A**) and left (**B**). In (**C**), Venn diagram on concordance of CSF cytology and dPCR.

Among the 109 right bone marrow samples analyzed, 24 samples were positive by dPCR, indicating expression of at least one of the target genes (Table S2). Of these, nine samples showed expression of both *RBP3* and *CRX*, and the presence of abnormal cells was confirmed by myelogram analysis (Figure 2A). Five samples showed expression of *RBP3* only, seven showed expression of *CRX* only, and three samples showed expression of both genes; all 15 of these samples were negative by myelogram analysis (Figure 2A). The remaining 85 samples were negative by both methods (Table S2).

For left BM, all six samples positive by myelogram were also positive by dPCR, with concurrent expression of both target genes (Figure 2B). Among the 102 myelogram-negative samples, 27 were dPCR-positive, including nine expressing *RBP3* only, 14 expressing *CRX* only and four expressing both genes (Figure 2B; Table S2).

Analysis of 110 CSF samples identified 11 samples positive for neoplastic cells by oncological cytology. Of these, two were positive by dPCR: one for both *RBP3* and *CRX* and one for *RBP3* only (Figure 2C). Among the 97 cytology-negative CSF samples, eight were positive by dPCR, including two positive for *RBP3* and six for *CRX* (Figure 2C; Table S2).

Using conventional diagnostic methods as the reference, dPCR showed a sensitivity of 100% and specificity of 90% for right BM, with positive and negative predictive values (PPV and NPV) of 37.5% and 100%, respectively. For left BM, sensitivity remained 100%, whereas specificity was 73.5%, with a PPV and an NPV of 18.2% and 100%, respectively. In CSF, dPCR demonstrated low sensitivity (15%) but high specificity (91.8%), with a PPV of 20% and an NPV of 89% (Table 2).

Together, these results indicate that a combined evaluation of both genes by dPCR was capable of identifying 249 samples, with a sensitivity and an NPV of 100%.

### 2.4. Detection of RBP3 and CRX Gene Expression in Pretreatment Samples

**Figure 3:**
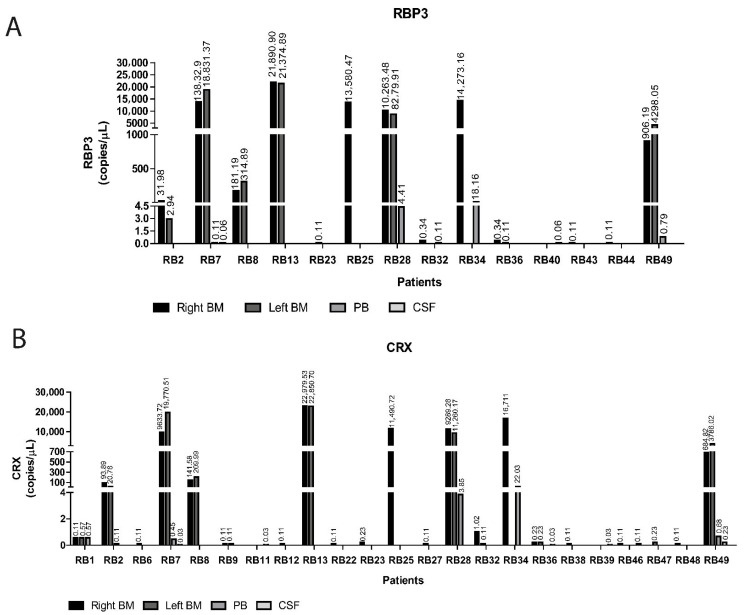
Expression of *RBP3* and *CRX* genes detected in 39 pretreatment samples from 22 patients. Expression of *RBP3* (**A**) and *CRX* (**B**) was assessed by digital PCR (dPCR) in pretreatment samples. Right BM—right bone marrow; Left BM—left bone marrow; PB—peripheral blood; CSF—cerebrospinal fluid.

Expression of *RBP3* and *CRX* was assessed by digital PCR (dPCR) in pretreatment samples from all 50 patients. In total, 191 samples were analyzed, including right BM (*n* = 49), left BM (*n* = 47), PB (*n* = 47) and CSF (*n* = 48). Expression of at least one target gene was detected in 48 samples from 28 patients (Figure 3).

Among the dPCR-positive samples, 22 showed expression of both genes, nine showed exclusive *RBP3* expression, and 17 showed exclusive *CRX* expression. Exclusive *RBP3* expression was observed in three patients, with concentrations ranging from 0.06 to 0.11 copies/µL (Figure 3A), whereas exclusive *CRX* expression was detected in 12 patients, with concentrations ranging from 0.03 to 0.58 copies/µL. Notably, patient RB1 showed consistent *CRX* expression across right BM, left BM and PB, with similar concentrations in all compartments (0.57–0.58 copies/µL) (Figure 3B). Patient RB9 showed *CRX* expression in left BM and PB at 0.11 copies/µL in both compartments (Figure 3B).

The remaining 11 patients showed expression of both *RBP3* and *CRX*, with concentrations ranging from 0.03 to 22,000 copies/µL. Patients RB2 and RB36 showed expression of both genes in right and left BM samples, as well as *CRX* expression in PB (RB2; 0.11 copies/µL) and CSF (RB36), respectively. Patient RB23 showed differential gene expression across compartments, with *RBP3* expression detected in left BM and *CRX* expression detected in right BM at concentrations of 0.11 and 0.23 copies/µL, respectively. Finally, patient RB32 showed expression of both genes in right BM, *CRX* expression only in left BM, and detection of *RBP3* in PB (Figure 3).

Overall, these findings demonstrate that dPCR enables sensitive detection of *RBP3* and *CRX* expression across multiple biological compartments at diagnosis, revealing heterogeneous dissemination patterns not captured by conventional assessments.

### 2.5. Detection of RBP3 and CRX Gene Expression in Samples Collected During Treatment

To evaluate minimal residual disease, longitudinal detection of the studied markers was performed during treatment in 22 of the 50 patients (44%) included in this study (Figure 1). Follow-up comprised between two and eight sampling time points per patient, depending on the clinical course (Table S3). Among these patients, four (RB3, RB14, RB17 and RB18) showed no detectable expression of *RBP3* or *CRX* at baseline or during treatment.

Among the remaining 18 patients (Supplementary Table S4), four (RB16, RB20, RB24 and RB26) were negative at the first sampling time point but showed detectable expression during treatment, with concentrations ranging from 0.11 to 0.23 copies/µL in BM samples (Supplementary Figure S4). In patient RB16, expression of both genes was also detected in PB (Supplementary Figure S4A,B).

**Figure 4:**
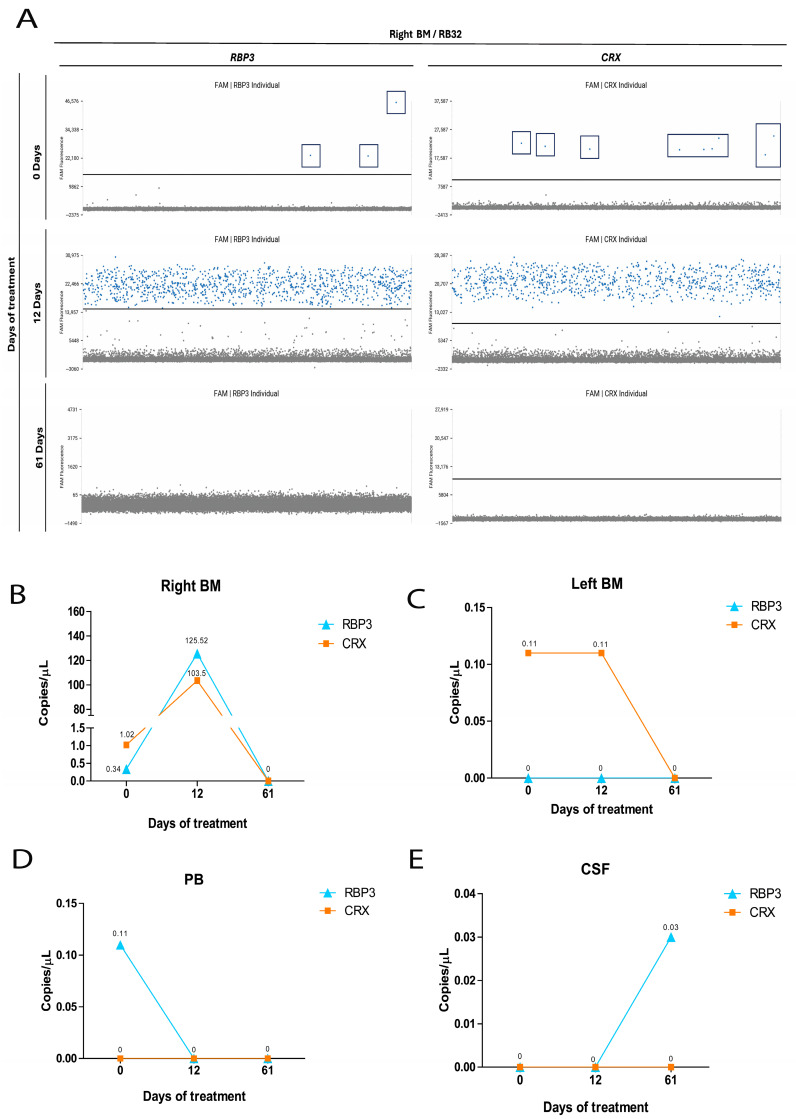
Detection of *RBP3* and *CRX* gene expression in samples collected during treatment in patient RB32. (**A**) Representative 1D scatter plots illustrating the detection of *RBP3* and *CRX* expression in patient RB32 during treatment. Distinct blue (FAM fluorescence) droplets indicate corresponding positive gene expression, and gray (VIC fluorescence) droplets indicate the absence of target amplification. Graphs showing the detection of gene expression during treatment from day 0 to day 61 in right bone marrow (right BM; (**B**)), left bone marrow (left BM; (**C**)), peripheral blood (PB; (**D**)), and cerebrospinal fluid (CSF; (**E**)).

Eigth patients (RB1, RB11, RB23, RB27, RB32, RB36, RB38 and RB43) were dPCR-positive at baseline despite negative myelogram and CSF cytology results (Table S1). In patient RB32, low-level expression of both genes (*RBP3*: 0.34 copies/µL; *CRX*: 1.02 copies/µL) was detected by dPCR in right BM at treatment initiation, with compartment-specific detection of *CRX* (0.11 copies/µL) in left BM and *RBP3* (0.11 copies/µL) in PB (Figure 4A–D). A follow-up sample collected 12 days after the first chemotherapy cycle showed increased *RBP3* (125.52 copies/µL) and *CRX* (103.5 copies/µL) copy numbers in right BM, concomitant with myelogram-confirmed BM infiltration (Figure 4A,B). Both assays became negative after the second chemotherapy cycle (day 61) (Figure 4). After 61 days of treatment, RBP3 expression was detected in the CSF, corroborating the cytological findings (Figure 4E).

The remaining six patients (RB7, RB8, RB13, RB25, RB28 and RB34) were positive by both myelogram and dPCR in BM samples at treatment initiation. At the second evaluation, BM and CSF samples were negative by conventional methods, consistent with treatment response (Table S1). In this context, dPCR detected low-level residual expression. Patients RB8 and RB34 showed *RBP3* expression in the left BM (0.11 copies/µL) after 102 and 21 days of treatment, respectively. Patients RB25 and RB28 showed isolated detection of *CRX* and *RBP3*, respectively, in the PB at the second evaluation (Table S4).

**Figure 5:**
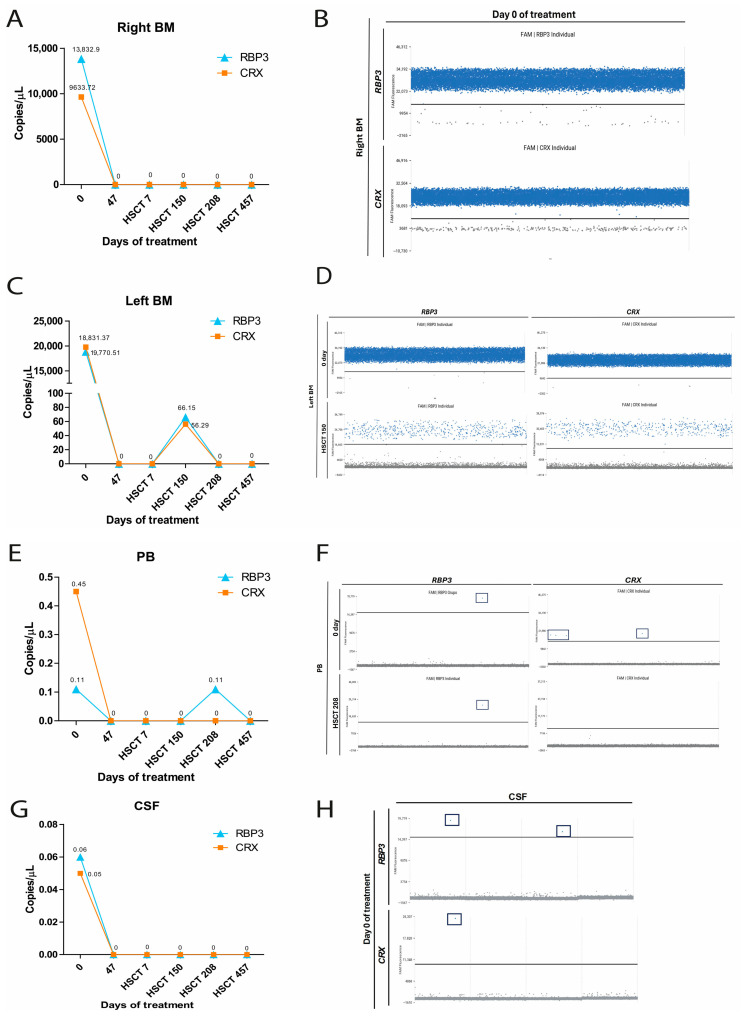
Detection of *RBP3* and *CRX* gene expression in samples collected during treatment in patient RB7. Graphs showing the detection of gene expression during treatment from day 0 to HSCT at 457 days in right bone marrow (right BM; (**A**)), left bone marrow (left BM; (**C**)), peripheral blood (PB; (**E**)), and cerebrospinal fluid (CSF; (**G**)). Representative 1D scatter plots illustrating the detection of *RBP3* and *CRX* expression in patient RB7 during treatment in right BM (**B**), left BM (**D**), PB (**F**), and CSF (**H**). Distinct blue (FAM fluorescence) droplets indicate corresponding positive gene expression, and gray (VIC fluorescence) droplets indicate the absence of target amplification.

**Figure 6:**
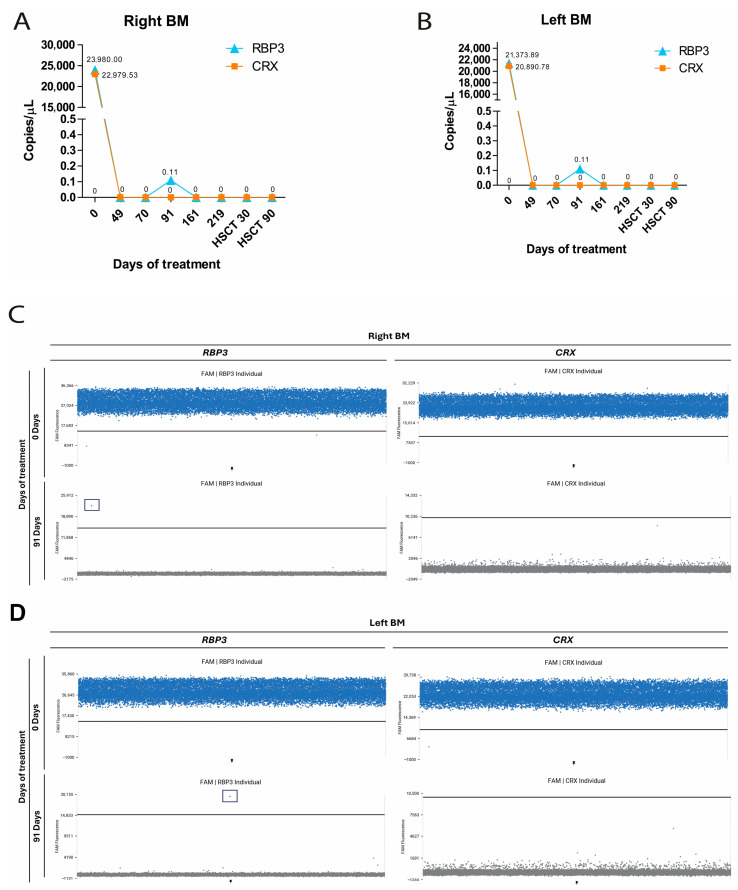
Detection of *RBP3* and *CRX* gene expression in samples collected during treatment in patient RB13. Graphs showing the detection of gene expression during treatment from day 0 to HSCT at 90 days in right BM (**A**) and left BM (**B**). Representative 1D scatter plots illustrating the detection of *RBP3* and *CRX* expression in patient RB32 during treatment in right BM (**C**) and left BM (**D**). Right BM—right bone marrow; Left BM—left bone marrow. Distinct blue (FAM fluorescence) droplets indicate corresponding positive gene expression, and gray (VIC fluorescence) droplets indicate the absence of target amplification.

Patients RB7 and RB13 had the longest follow-up (495 and 410 days, respectively) and underwent chemotherapy followed by autologous hematopoietic stem cell transplantation. At baseline, both showed high expression of *RBP3* and *CRX* in bilateral BM (Figure 5A–D and Figure 6), and patient RB7 also showed positivity in PB and CSF, consistent with myelogram and cytology findings (Figure 5 E–H). After two chemotherapy cycles, both patients became negative by dPCR. At later time points, re-emergence of *RBP3* and *CRX* expression was detected in the left BM of patient RB7 at day 287 (150 days post transplant), followed by low-level *RBP3* detection at the final follow-up (Figure 5). Patient RB13 remained largely negative, with only transient low-level *RBP3* expression detected at day 91 (Figure 6).

Longitudinal dPCR analysis enabled sensitive detection of dynamic changes in *RBP3* and *CRX* expression during treatment, frequently preceding or persisting beyond conventional diagnostic findings, thereby supporting its utility for monitoring minimal residual disease and early relapse in retinoblastoma.

## 3. Discussion

In retinoblastoma, early identification of disease dissemination is of critical importance, as it enables treatment intensification and may improve outcomes for patients with advanced disease. Tumor dissemination may involve the central nervous system, including the brain and CSF, as well as hematogenous spread to the BM. The use of liquid biopsy for the detection of minimal disseminated disease has enabled real-time tumor monitoring and the identification of molecular markers in the absence of tumor tissue samples [14,22,28,29,30].

In this study, the potential of liquid biopsy as an approach for molecular monitoring of retinoblastoma was demonstrated by assessing *CRX* and *RBP3* gene expression in ctcRNA (circulating tumor cell RNA) and cfRNA. These markers were detected in BM, CSF, and PB samples using digital PCR (dPCR). The findings reinforce the relevance of *CRX* and *RBP3* as highly specific markers of retinal tumor cells. Moreover, the integration of molecular data with conventional diagnostic methods highlights clear advantages of the molecular approach in settings of low tumor burden, as well as its potential for dynamic disease monitoring throughout treatment. The results highlight both the strengths and compartment-specific limitations of cfRNA-based detection [21,22].

To our knowledge, this is the first study to evaluate 433 samples—including bilateral BM, PB, and CSF—from a cohort of 50 patients using dPCR for the detection of minimal disseminated disease. Bilateral BM sampling is commonly employed to enhance detection sensitivity, given that tumor infiltration may occur in a focal and heterogeneous manner. Within this cohort, 82% of patients were classified as having Group E retinoblastoma, representing the most advanced stage of disease according to the International Intraocular Retinoblastoma Classification (IIRC) [31].

Comparison of dPCR results with myelogram demonstrated strong concordance in BM samples, with 100% sensitivity and negative predictive value for both right and left BM, and specificities of 87.1% and 75.9% for the right and left BM, respectively. All samples positive by myelogram were consistently detected by dPCR, with concomitant expression of *CRX* and *RBP3*, supporting the robustness of these markers for identifying BM involvement. Notably, a substantial proportion of BM samples that were negative on myelogram showed detectable gene expression by dPCR, thereby reducing the specificity and positive predictive value. This discrepancy likely reflects the superior analytical sensitivity of dPCR, enabling detection of tumor-derived transcripts below the threshold of cytomorphological recognition. Such findings suggest that molecular positivity may precede overt cytological evidence of marrow infiltration, highlighting the potential role of dPCR in identifying early or minimal disease dissemination.

The present findings highlight important differences in diagnostic performance across biological samples. In contrast to peripheral blood and bone marrow ctcRNA analyses, cfRNA detection in cerebrospinal fluid (CSF) exhibited a distinct diagnostic profile. While digital PCR (dPCR) demonstrated high specificity (92.4%), sensitivity was low (18.2%) when compared with oncological cytology. These results indicated that the presence of *CRX* or *RBP3* cfRNA in CSF was highly specific for central nervous system involvement, whereas the absence of detectable transcripts did not exclude disease. This limited sensitivity was likely attributable to biological factors intrinsic to the CSF compartment, including low tumor cell shedding, dilution effects, and rapid cfRNA degradation. Nevertheless, the high specificity suggested that positive cfRNA detection in CSF carried substantial confirmatory value.

Consistent with this interpretation, previous studies have demonstrated that the sensitivity of CSF-based liquid biopsy approaches depends on tumor burden, anatomical proximity to CSF spaces, and the presence of a direct tumor–CSF interface. Across both central nervous system (CNS) and non-CNS malignancies, detection rates have been shown to decrease substantially in low-shedding or compartmentalized tumors, whereas specificity remains consistently high [32,33,34].

Taken together, these observations suggest that CSF cfRNA analysis may have a limited role as a screening tool but may provide clinically meaningful confirmatory evidence when positive, particularly in cases with suspected central nervous system involvement.

Pretreatment analysis revealed detectable expression of *CRX* and/or *RBP3* in nearly half of the patients, frequently at very low transcript concentrations. These findings emphasize the biological heterogeneity of retinoblastoma dissemination and support the combined assessment of both markers to enhance detection sensitivity. Cases such as RB1 and RB32 demonstrate that dPCR can detect early multicompartmental involvement, preceding findings that become evident only later. Studies have demonstrated the importance of investigating and monitoring patients with RB who present high-risk pathological features, following the analysis of CSF and BM samples from patients with RB [22]. *CRX* expression was assessed by real-time PCR, and minimal disease was detected in nine of 96 patients, four of whom subsequently developed metastatic relapse [23]. These results highlight the importance of investigating and longitudinally monitoring patients with RB who exhibit high-risk pathological features. Notably, the detection of ctcRNA in PB and cfRNA CSF in selected patients, even in the absence of cytological evidence, reinforces the concept that tumor-derived RNA fragments circulate systemically and can be captured through sensitive molecular approaches.

Longitudinal cfRNA and ctcRNA analysis during treatment provided further evidence of the clinical relevance of this strategy for minimal residual disease (MRD) monitoring. Additional technical challenges in liquid biopsy analysis include variability in pre-analytical and analytical conditions, which can limit the reproducibility and clinical implementation of circulating tumor cell (CTC) and circulating tumor DNA/RNA platforms for the detection and characterization of minimal residual disease [35].

In several patients, dPCR detected *CRX* and *RBP3* expression despite negative myelogram and CSF cytology at baseline, with subsequent increases in transcript levels preceding cytological confirmation of BM infiltration. Conversely, clearance of cfRNA signals consistently accompanied cytological remission following chemotherapy. These observations suggest that cfRNA dynamics closely mirror tumor burden and may precede detectable morphological changes, offering a window for earlier intervention.

Extended follow-up in selected patients further highlighted the potential role of cfRNA and ctcRNA monitoring in long-term surveillance. Re-emergence of *CRX* and *RBP3* expression in BM after hematopoietic stem cell transplantation, in some cases at high copy numbers, suggested molecular relapse or persistent subclinical disease. The detection of low-level ctcRNA expression at later time points underscores the sensitivity of dPCR and raises important questions regarding the prognostic significance of minimal molecular positivity, which warrants further investigation.

Taken together, these findings demonstrate that ctcRNA- and cfRNA-based liquid biopsy using *CRX* and *RBP3* provides a sensitive and dynamic measure of disseminated retinoblastoma, particularly in BM. Future studies in larger, multicentric cohorts will be essential to define standardized thresholds, clarify the prognostic significance of low-level positivity, and determine how this approach can be optimally incorporated into risk stratification, treatment monitoring, and long-term follow-up of patients with retinoblastoma.

## 4. Methods

### 4.1. Patients and Sample Collection

A total of 433 samples, including bone marrow (BM), peripheral blood (PB) and cerebrospinal fluid (CSF), were collected from 50 patients with retinoblastoma treated at the Pediatric Oncology Institute–Grupo de Apoio ao Adolescente e à Criança com Câncer, Federal University of São Paulo (IOP-GRAACC/UNIFESP). Two patients were included retrospectively, and 48 patients were prospectively enrolled in 2024. Samples from all 50 patients were obtained at baseline, before the initiation of chemotherapy, and samples from 22 patients were subsequently obtained after every two chemotherapy cycles. The study was conducted in accordance with the Declaration of Helsinki and approved by the Institutional Research Ethics Committee of the Federal University of São Paulo (approval no. 0715P/2021). All samples belong to the Pediatric Oncology Institute Biobank IOP/GRAACC/UNIFESP (National Commission of Ethics in Research—CONEP B-053). Informed consent to participate in the study was obtained from participants (or their parent or legal guardian for children under the age of 17).

### 4.2. Cerebrospinal Fluid (CSF) Cell-Free RNA (cfRNA) Extraction

Approximately 2.0 mL of cerebrospinal fluid (CSF) was collected per patient and processed immediately. Samples were centrifuged at 2000× *g* for 10 min at 4 °C, and the resulting supernatants were further centrifuged at 1400× *g* for 10 min at 4 °C to remove residual cellular debris. Cell-free RNA (cfRNA) was extracted from CSF supernatants using the QIAamp ccfDNA/RNA Kit (Qiagen, Hilden, Germany), according to the manufacturer’s instructions, with a final elution volume of 100 µL. Genomic DNA was removed using gDNA Wipeout Buffer (QuantiTect Reverse Transcription Kit, Qiagen) following the manufacturer’s protocol. cfRNA concentration was quantified using the QuantiFluor^®^ RNA System on a Quantus Fluorometer (Promega, Madison, WI, USA).

### 4.3. RNA Extraction from Bone Marrow (BM) and Peripheral Blood (PB)

BM (right and left) and PB, 2 mL each, were extracted using the AllPrep DNA/RNA Mini Kit (Qiagen), as described by the manufacturer. RNA was quantified using the Nanodrop 2000 and Qubit 3 Fluorometer (Thermo Fisher Scientific).

### 4.4. Assessment of CRX and RBP3 Expression Using Digital PCR (dPCR)

Expression of CRX and RBP3 was assessed using digital PCR (dPCR) on the QuantStudio™ Absolute Q™ Digital PCR System (Thermo Fisher Scientific). Reactions were prepared using Absolute Q™ 1-Step RT-dPCR Master Mix (4×; Applied Biosystems™), TaqMan^®^ Gene Expression Assays for *RBP3* (Hs00161253_m1) and *CRX* (Hs00230899_m1) labeled with FAM, and *ACTB* (Hs01060665_g1) labeled with VIC (Thermo Fisher Scientific, Waltham, MA, USA). A total of 50 ng of RNA from BM and PB samples, or 5 µL of total cell-free RNA from CSF samples, was added per reaction. Reaction mixtures were loaded into QuantStudio™ Absolute Q™ MAP16 plates and automatically partitioned into 20,480 microchambers per well. Thermal cycling was performed as follows: 96 °C for 10 min, followed by 40 cycles of 96 °C for 5 s and 60 °C for 15 s.

To increase assay sensitivity for cerebrospinal fluid samples, each sample was analyzed in quadruplicate, thereby increasing the total number of microchambers assessed. Fluorescence thresholds for FAM and VIC were determined following assay standardization. Data were analyzed using QuantStudio™ Absolute Q™ Digital PCR Software (v6.3.5).

All reactions included appropriate controls, comprising positive controls (known positive samples) and negative controls, consisting of bone marrow and peripheral blood samples obtained from healthy donors, in addition to no-template controls (reaction mix without sample).

Data obtained by digital PCR were compared with the results of bone marrow smear examination and CSF analysis for the detection of neoplastic cells.

### 4.5. dPCR Assay Standardization

The sensitivity of the dPCR assay for assessing *CRX* and *RBP3* expression was evaluated using a concentration gradient (20%, 10%, 1%, 0.1%, 0.01% and 0.001%) of RNA extracted from the Y79 retinoblastoma cell line (ATCC HTB-18), which exhibits high expression of the target genes. Serial dilutions were generated by mixing Y79 RNA with RNA from the KHOS mesenchymal osteosarcoma cell line (ATCC CRL-1546), which lacks expression of the genes of interest. In addition, two BM RNA samples from patients with retinoblastoma with confirmed BM invasion by tumor cells were included as positive biological controls.

### 4.6. Statistical Analysis

Statistical analysis was performed using GraphPad Prism version 7.0 (GraphPad Software, San Diego, CA, USA) for Windows. Linear regression analysis was applied to evaluate the relationship between expected and observed values in the limit of detection (LoD) experiments. The goodness of fit was assessed by the coefficient of determination (R^2^).

The characteristics tested were the sensitivity, specificity, positive predictive value (PPV), negative predictive value (NPV). Sensitivity was defined as the proportion of true positives (TPs) among all actual positive cases (TPs + false negatives [FNs]), whereas specificity was defined as the proportion of true negatives (TNs) among all actual negative cases (TNs + false positives [FPs]). The PPV was calculated as the proportion of true positives among all positive test results (TPs + FPs), and the NPV was calculated as the proportion of true negatives among all negative test results (TNs + FNs). Accuracy was defined as the proportion of correctly classified cases among the total number of samples analyzed.

## Figures and Tables

**Table 1:** Clinical demographics of 50 patients with retinoblastoma included in the study.

	Retinoblastoma			
	Unilateral	Bilateral	Trilateral	All
**Number of patients (%)**	30 (60%)	17 (34%)	3 (6%)	50 (100%)
**Mean age at diagnosis (months)**	29	20	26	26
**Gender**				
Male	15 (50%)	10 (59%)	0	25 (50%)
Female	15(50%)	7 (41%)	3 (100%)	25(50%)
**Tumor**				
Intraocular	19 (63%)	9 (53%)	1 (33%)	29 (58%)
Extraocular	11 (37%)	8 (47%)	2 (67%)	21 (42%)
**Primary treatment**				
Enucleation	7 (23%)	1 (6%)	0	8 (16%)
Therapy	23 (77%)	16 (94%)	3 (100%)	42 (84%)
**Enucleation**				
Yes	24 (80%)	12 (71%)	2 (67%)	38 (76%)
No	6 (20%)	5 (29%)	1 (33%)	12 (24%)
**Recurrence**	4 (13%)	6 (35%)	0	10 (20%)
**Mortality**	3 (10%)	2 (12%)	1 (33%)	6 (12%)
**TCTH**	6 (20%)	2 (12%)	0	8 (16%)
**IIRC group (70 eyes)**				
A	0	3 (9%)	0	3 (4%)
B	0	0	0	0
C	1 (3%)	5 (14%)	0	6 (8%)
D	0	7 (21%)	1 (17%)	8 (11%)
E	27 (90%)	19 (56%)	3 (50%)	49 (70%)
Without group	2 (7%)	0	2 (33%)	4 (6%)

**Table 2:** Overall diagnostic performance of digital PCR assay and myelogram and CSF cytology analysis for 433 samples. Right BM: right bone marrow; Left BM: left bone marrow; CSF: cerebrospinal fluid; PB: peripheral blood; PPV: positive predictive value; NPV: negative predictive value.

Sample	Sensitivity	Specificity	PPV	NPV
**Right BM**	100%	90%	37.5%	100%
**Left BM**	100%	73.5%	18.2%	100%
**CSF**	15%	91.8%	20%	89%

## Data Availability

All data generated or analyzed during this study are included within the article.

## Supplementary Materials

The following supporting information can be downloaded at: https://www.mdpi.com/article/10.3390/ijms27104177/s1.
