# Supplementary material for: Digital PCR Quantification of a Circulating RBP3 and CRX RNA Signature Establishes a Liquid Biopsy Framework for Precision Monitoring of Retinoblastoma
Source: Int J Mol Sci. 2026 May 8;27(10):4177. doi: 10.3390/ijms27104177 (PMC13206994; doi:10.3390/ijms27104177)
Supplement: Supplementary file 1 [file ijms-27-04177-s001.zip › Supplementary Table S4.pdf]

Supplementary Table S4: Results of *RBP3* and *CRX* gene expression assessed by digital PCR in samples from the 18 patients evaluated during treatment.

Patient	Days of treatment	Sample	*RBP3* Conc. Cp/µL	*ACTB* Conc. Cp/µL	*CRX* Conc. Cp/µL	*ACTB* Conc. Cp/µL
RB1	0	RIGHT-BM	0	10254.25	0.58068	14747.29
RB1	0	LEFT-BM	0	3739.21	0.57	19770.51
RB1	0	PB	0	7144.93	0.57	7664.11
RB1	0	CSF	0	0	0	0
RB1	481	RIGHT-BM	1.49	9175.12	2.71	9354.17
RB1	481	LEFT-BM	4.03	10971.48	13.6	22979.64
RB1	481	PB	0	15822.75	0	15721.55
RB1	481	CSF	0	0.34	0	0.08
RB1	595	RIGHT-BM	0	7462.89	0.11	8386.01
RB1	595	LEFT-BM	0	4386.56	0.68	3378.53
RB1	595	PB	0	13789.04	0.12	11118.35
RB1	HSCT 43	LEFT-BM	0	22976.92	0	21372.08
RB1	HSCT 43	PB	0	22979.64	0	5837.29
RB7	0	RIGHT-BM	13832.9	10579,79	9633.72	18474.65
RB7	0	LEFT-BM	18831.37	9752,73	19770.51	18166
RB7	0	PB	0.11	14273.16	0.45	12589.05
RB7	0	CSF	0.06	0.11	0.055	0.285
RB7	47	RIGHT-BM	0	22979.41	0	22979.53
RB7	47	LEFT-BM	0	22979.64	0	22979.64
RB7	47	PB	0	22979.64	0	22979.64
RB7	47	CSF	0	0.11	0	0
RB7	HSCT 47	RIGHT-BM	0	22976.2	0	22976.02
RB7	HSCT 47	LEFT-BM	0	22979.53	0	22979.64
RB7	HSCT 47	PB	0	5417.59	0	2830.22
RB7	HSCT 47	CSF	0	0	0	0
RB7	HSCT 150	RIGHT-BM	0	17419.8	0	17419.8
RB7	HSCT 150	LEFT-BM	66.15	19254.09	56.29	22979.64
RB7	HSCT 150	PB	0	11412.06	0	10894.88
RB7	HSCT 150	CSF	0	0.08	0	0
RB7	HSCT 208	RIGHT-BM	0	22978.17	0	22978.40
RB7	HSCT 208	LEFT-BM	0	22979.64	0	22979.64
RB7	HSCT 208	PB	0.11	22979.64	0	22979.64
RB7	HSCT 208	CSF	0	0	0	0
RB7	HSCT 300	RIGHT-BM	0	22937.67	0	22979.64
RB7	HSCT 300	LEFT-BM	0	22979.64	0	22979.53
RB7	HSCT 300	PB	0	22979.64	0	22979.64
RB7	HSCT 300	CSF	0	0.42	0	1.3
RB7	HSCT 360	RIGHT-BM	0	22978.85	0	22978.96
RB7	HSCT 360	LEFT-BM	0	22978.96	0	22978.51
RB7	HSCT 360	PB	0	0.11	0	0.79

		CSF	0	0.87	0	0.48
	HSCT 457	RIGHT-BM	0	17421.14	0	14950.42
		LEFT-BM	0	22979.41	0	22979.41
		PB	0	22977.94	0	19768.59
		CSF	0	36.8	0	33.99
RB8	0	RIGHT-BM	181.19	22978.28	141.58	22977.83
		LEFT-BM	314.89	22978.96	209.99	22979.41
		PB	0	22979.64	0	22952.81
		CSF	0	0	0	0
	102	RIGHT-BM	0	22979.53	0	22979.64
		LEFT-BM	0.11	22979.64	0	22979.64
		PB	0	22979.64	0	18831.93
		CSF	0	0.03	0	0.08
	199	RIGHT-BM	0	21375.13	0	22979.64
		LEFT-BM	0	22974.77	0	22975.79
		PB	0	22979.64	0	22979.64
		CSF	0	0	0	0.1
RB11	0	RIGHT-BM	0	22979.53	0	22946.86
		LEFT-BM	0	22979.41	0	11896.69
		PB	0	19770.62	0	11840.32
		CSF	0	0.06	0.03	0
	23	CSF	0.03	0	0	0
	44	CSF	0	0	0	0
	115	RIGHT-BM	0	22931.67	0	22979.53
		LEFT-BM	0	22979.64	0	22979.41
		PB	0	201375	0	22979.64
		CSF	0	0.03	0	0
	245	RIGHT-BM	0	22979.53	0	22925.78
		LEFT-BM	0	22979.64	0	19770.4
		PB	0	0.45	0	19770.6
		CSF	0	3.57	0	0.03
	329	RIGHT-BM	0	3.57	0	0.03
		LEFT-BM	0	22952.12	0.11	22979.41
		PB	0	22978.96	0	22989.64
		CSF	0	22979.64	0	22979.53
	401	RIGHT-BM	0.11	0	0	0
		LEFT-BM	0	22974.89	0	22976.25
		PB	0	22978.06	0	22979.41
		CSF	0	22978.85	0	22979.19
	490	RIGHT-BM	0	0	0	0
		LEFT-BM	0	22978.96	0.11	22979.19
		PB	0	14614.92	0	14493.40
		CSF	0	22977.38	0	22977.38

RB13	0	RIGHT-BM	21890.90	22979.64	22979.53	21373.89
		LEFT-BM	21373.89	21373.89	22850.70	22979.64
	49	RIGHT-BM	0	22979.30	0	22979.19
		LEFT-BM	0	22979.64	0	22979.64
		PB	0	22979.64	0	22979.64
		CSF	0	0	0	0
	70	RIGHT-BM	0	22979.41	0	22979.41
		LEFT-BM	0	22979.64	0	22933.29
		PB	0	22923.23	0	22979.64
		CSF	0	0	0	0
	91	RIGHT-BM	0.11	21374.79	0	22972.39
		LEFT-BM	0.11	22979.53	0	20436.56
		PB	0	22979.64	0	20436.5
		CSF	0	0	0	0
	161	RIGHT-BM	0	22979.07	0	22950.29
		LEFT-BM	0	22958.98	0	22929.48
		PB	0	19254.09	0	17893.36
		CSF	0	0	0	0
	219	RIGHT-BM	0	22979.64	0	22979.64
		LEFT-BM	0	22977.94	0	22977.26
		PB	0	22977.94	0	22978.28
		CSF	0	0.99	0	1.39
	HSCT 30	RIGHT-BM	0	22976.36	0	22973.19
		LEFT-BM	0	22978.73	0	22978.85
		PB	0	22978.73	0	22979.53
		CSF	0	0	0	0
	HSCT 90	RIGHT-BM	0	22978.62	0	22979.41
		LEFT-BM	0	16283.53	0	21369.47
		PB	0	22978.17	0	22976.25
		CSF	0	0	0	0.08
RB16	0	RIGHT-BM	0	22970.69	0	22966.60
		LEFT-BM	0	22936.17	0	22969.90
		PB	0	22797.64	0	22979.64
		CSF	0	0.06	0	0
	51	RIGHT-BM	0	22979.64	0	22979.53
		LEFT-BM	0	22979.64	0	22979.64
		PB	0.11	22979.64	0.23	22979.64
		CSF	0	0	0	0
	72	RIGHT-BM	0	22979.64	0	22948.80
		LEFT-BM	0	22978.85	0	22960.12
		PB	0	22954.30	0	22979.38
		CSF	0	0	0	0
RB20	0	RIGHT-BM	0	22975.23	0	22951.55

		LEFT-BM	0	22940.77	0	22979.53
		PB	0	22979.53	0	21375.13
		CSF	0	0	0	0
	47	RIGHT-BM	0	22965.69	0	22979.41
		LEFT-BM	0	22977.26	0	22979.64
		PB	0	387.20	0	3142.89
		CSF	0	0	0	0
	87	RIGHT-BM	0	473.5	0	213.7
		LEFT-BM	0	3821.03	0	22977.94
		PB	0	4939.86	0	1800.94
		CSF	0	0	0	0
	195	RIGHT-BM	0	22978.17	0.11	22978.73
		LEFT-BM	0	22979.07	0	22979.07
		PB	0	22979.64	0	22979.53
		CSF	0	0	0	0
RB23	0	RIGHT-BM	0	22954.52	0.23	22955.67
		LEFT-BM	0.11	22970.35	0	22978.51
		PB	0	22977.38	0	22977.94
		CSF	0	5.55	0	9.96
	153	RIGHT-BM	0	22979.07	0	22979.64
		LEFT-BM	0	22979.41	0	22979.41
		PB	0	21375.13	0	22979.64
		CSF	0	0.73	0	0.48
RB24	0	RIGHT-BM	0	22979.41	0	22979.41
		LEFT-BM	0	22956.12	0	22941.81
		PB	0	22936.05	0	22979.64
		CSF	0	0	0	0
	21	RIGHT-BM	0	22952.81	0	22952.81
		LEFT-BM	0	22962.96	0	22973.07
		PB	0	22979.64	0	22974.21
		CSF	0	0.73	0	0.48
	42	RIGHT-BM	0	22959.32	0	22955.78
		LEFT-BM	0	22947.20	0	22964.67
		PB	0	22930.63	0	22979.53
		CSF	0	0	0	0
	77	RIGHT-BM	0	22972.39	0	22972.39
		LEFT-BM	0	22977.49	0	22977.49
		PB	0	22978.17	0	17888.38
		CSF	0	0	0	0
	216	RIGHT-BM	0	22978.17	0	22978.51
		LEFT-BM	0.11	22979.30	0	22978.96
		PB	0	22977.94	0	22976.81
		CSF	0	0	0	0

RB25	0	RIGHT-BM	13580.47	22979.64	11490.72	22979.64
		CSF	0	0	0	0
	45	RIGHT-BM	0	22975.68	0	22978.17
		LEFT-BM	0	22975.34	0	22976.13
		PB	0	22977.49	0.23	22977.38
		CSF	0	0	0	0
	66	RIGHT-BM	0	22977.83	0	22954.52
		LEFT-BM	0	22979.41	0	22979.64
	179	RIGHT-BM	0	22977.49	0	22978.17
		LEFT-BM	0	21372.64	0	18468.09
		PB	0	18832.05	0	13245.08
		CSF	0	0	0	0
	240	RIGHT-BM	0	22977.38	0	22978.73
		LEFT-BM	0.11	13023.82	0	20431.92
		PB	0	13245.08	0	9463.43
		CSF	0	11.01	0	0
RB26	0	RIGHT-BM	0	20436.33	0	22977.83
		LEFT-BM	0	22975.79	0	22952.81
		PB	0	475.8	0	3142.89
		CSF	0	0.03	0	0.06
	76	RIGHT-BM	0	22979.64	0	22975.79
		LEFT-BM	0	22979.64	0.11	22979.64
		PB	0	22952.81	0	22974.77
		CSF	0	0	0	0
	104	RIGHT-BM	0	22979.64	0	22952.81
		LEFT-BM	0	22977.83	0	22974.77
		PB	0	22979.64	0	22979.64
		CSF	0	6.26	0	10.38
	139	RIGHT-BM	0	22975.79	0.11	22952.81
		LEFT-BM	0	22979.64	0	22977.83
		PB	0	22979.53	0	22979.64
		CSF	0	0	0	0
RB27	0	RIGHT-BM	0	22978.17	0	22978.40
		LEFT-BM	0	22937.67	0.11	22979.64
		PB	0	22979.64	0	22979.64
		CSF	0	0	0	0
	96	RIGHT-BM	0	22978.40	0	22978.17
		LEFT-BM	0	22978.17	0	22978.40
		PB	0	18832.05	0	17649.58
		CSF	0	0	0	0
RB28	0	RIGHT-BM	10263.48	22979.64	11260.17	22979.41
		LEFT-BM	8729.91	22252.64	9289.28	22459.52
		PB	4.41	19770.51	3.85	19785.51

		CSF	0	0	0	0
	49	RIGHT-BM	0	22978.17	0	22976.20
		LEFT-BM	0	22976.42	0	22979.64
		PB	0.11	19770.51	0	19770.54
		CSF	0	1.44	0	2.29
	118	CSF	0	0	0	0
	198	RIGHT-BM	0	22979.64	0	22975.25
		LEFT-BM	0	22979.64	0	22978.17
		PB	0	22979.64	0	22949.68
		CSF	0	0	0	0
	213	CSF	0	11.58	0	33.69
RB32	0	RIGHT-BM	0.34	21372.64	1.02	19251.49
		LEFT-BM	0	19768.93	0.11	18829.79
		PB	0.11	15349.48	0	22978.62
		CSF	0	0	0	0
	12	RIGHT-BM	125.52	14016.49	103.5	19769.27
		LEFT-BM	0	15528.42	0.11	15437.75
		PB	0	19254.09	0	22979.64
		CSF	0	0	0	0
	61	RIGHT-BM	0	21375.13	0	18166.12
		LEFT-BM	0	11996	0	11275.37
		PB	0	22979.64	0	22978.40
		CSF	0.03	0.03	0	0
RB34	0	RIGHT-BM	14273.16	22979.52	16711	22979.53
		PB	18.16	22256.63	22.03	22359.58
		CSF	0	0.11	0	0
	21	RIGHT-BM	0	22979.53	0	22979.64
		LEFT-BM	0.11	22979.64	0	22975.25
		PB	0	14747.29	0	14758.29
		CSF	0	0	0	0
	42	RIGHT-BM	0	22975.79	0	22985.79
		LEFT-BM	0	22975.79	0	22976.13
		PB	0	19770.51	0	22978.28
		CSF	0	0	0	0
	78	RIGHT-BM	0	22979.64	0	22479.64
		LEFT-BM	0	22855.64	0	22979.64
RB36	0	RIGHT-BM	0.34	22979.64	0.23	22979.53
		LEFT-BM	0.11	20374.11	0.23	20978.17
		PB	0	10894.88	0	11412.06
		CSF	0	1.64	0.03	1.3
	68	RIGHT-BM	0	22979.64	0	22979.64
		LEFT-BM	0	21366.75	0	18468.09
		PB	0	22978.17	0	22224.86

		CSF	0	40.4	0.11	27.75
	90	RIGHT-BM	0	22923.64	0	22979.64
		LEFT-BM	0.23	22979.64	0.11	22879.45
		PB	0	22979.53	0	22949.53
		CSF	0	4.31	0	5.33
RB38	0	BM Right	0	22979.64	0	22976.92
		BM Left	0	22975.79	0.11	22979.64
		PB	0	19770.51	0	22976.92
		CSF	0	0	0	0
	119	BM Right	0	22979.64	0	22979.41
		BM Left	0.11	22976.92	0	22975.79
		PB	0	19770.51	0	18831.93
		CSF	0	0	0	0
RB43	0	RIGHT-BM	0.11	22979.64	0	22976.2
		LEFT-BM	0	22937.67	0	22979.53
		PB	0	22979.64	0	22978.17
		CSF	0	0	0	0
	46	RIGHT-BM	0.11	22978.40	0	22979.53
		LEFT-BM	0.11	22978.40	0.11	22937.67
		PB	0	22979.64	0	22979.53
		CSF	0	0	0.06	1
	74	RIGHT-BM	0	22979.64	0	22937.67
		LEFT-BM	0	22937.67	0	22979.41
		PB	0	10894.88	0	11412.06
		CSF	0	0	0.06	1

Right-BM: Right bone marrow; Left-BM: Left bone marrow; CSF: cerebrospinal fluid; PB: peripheral blood.
