# Supplementary material for: Digital PCR Quantification of a Circulating RBP3 and CRX RNA Signature Establishes a Liquid Biopsy Framework for Precision Monitoring of Retinoblastoma
Source: Int J Mol Sci. 2026 May 8;27(10):4177. doi: 10.3390/ijms27104177 (PMC13206994; doi:10.3390/ijms27104177)
Supplement: Supplementary file 1 [file ijms-27-04177-s001.zip › Supplementary Figure S1.pdf]

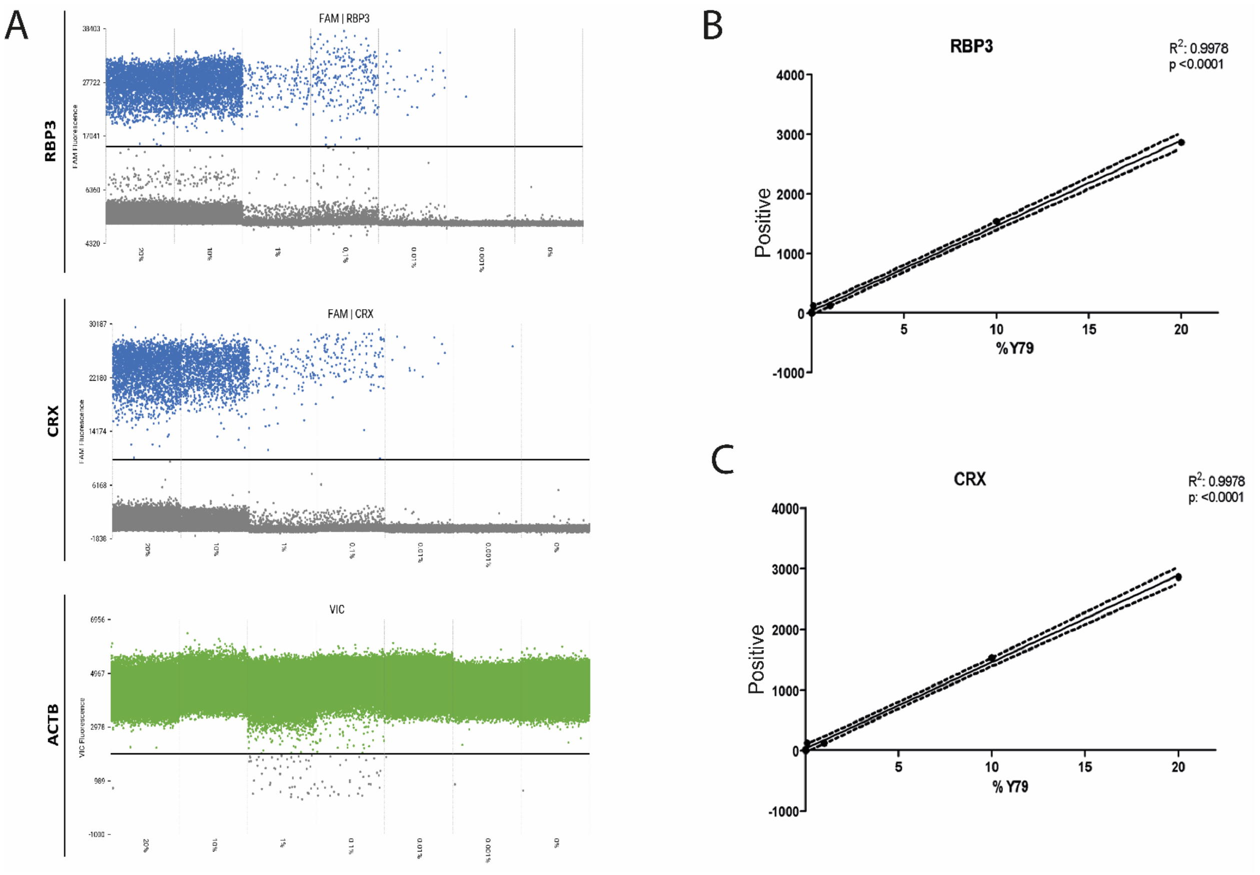

Supplementary Figure S1: Representation of *RBP3* and *CRX* gene expression across the Y79 cell line dilution series using the QuantStudio Absolute Q™ Digital PCR Software. Schematic overview of the 1D scatter plots (A). Correlation plots of positive microchambers for *RBP3* (B) and *CRX* (C) according to the Y79 dilution curve. The curves display the correlation coefficient (R^2) and statistically significant p values ($p < 0.0001$). Distinct blue (FAM fluorescence) droplets indicate corresponding gene expression positive and the gray (VIC fluorescence) droplets indicate the absence of target amplification
