# Supplementary material for: Digital PCR Quantification of a Circulating RBP3 and CRX RNA Signature Establishes a Liquid Biopsy Framework for Precision Monitoring of Retinoblastoma
Source: Int J Mol Sci. 2026 May 8;27(10):4177. doi: 10.3390/ijms27104177 (PMC13206994; doi:10.3390/ijms27104177)
Supplement: Supplementary file 1 [file ijms-27-04177-s001.zip › Supplementary Figure S3.pdf]

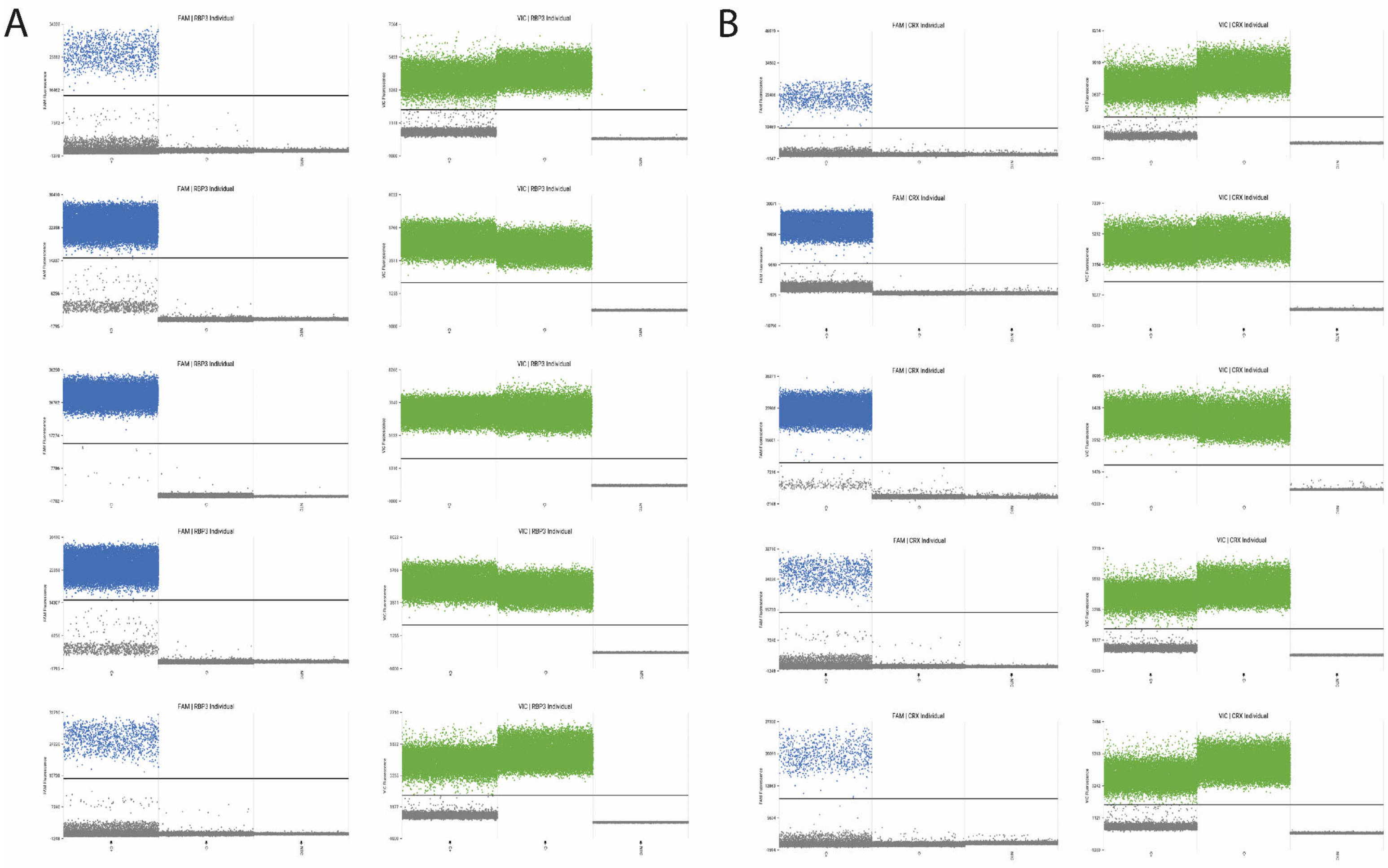

Supplementary Figure S3: Representation of 1D scatter plots of *RBP3* and *CRX* gene expression in positive (C+) and negative (C−) control samples, demonstrating robust assay performance and reproducibility. Experiments were performed in quintuplicate.
