# Supplementary material for: Digital PCR Quantification of a Circulating RBP3 and CRX RNA Signature Establishes a Liquid Biopsy Framework for Precision Monitoring of Retinoblastoma
Source: Int J Mol Sci. 2026 May 8;27(10):4177. doi: 10.3390/ijms27104177 (PMC13206994; doi:10.3390/ijms27104177)
Supplement: Supplementary file 1 [file ijms-27-04177-s001.zip › Supplementary Figure S4.pdf]

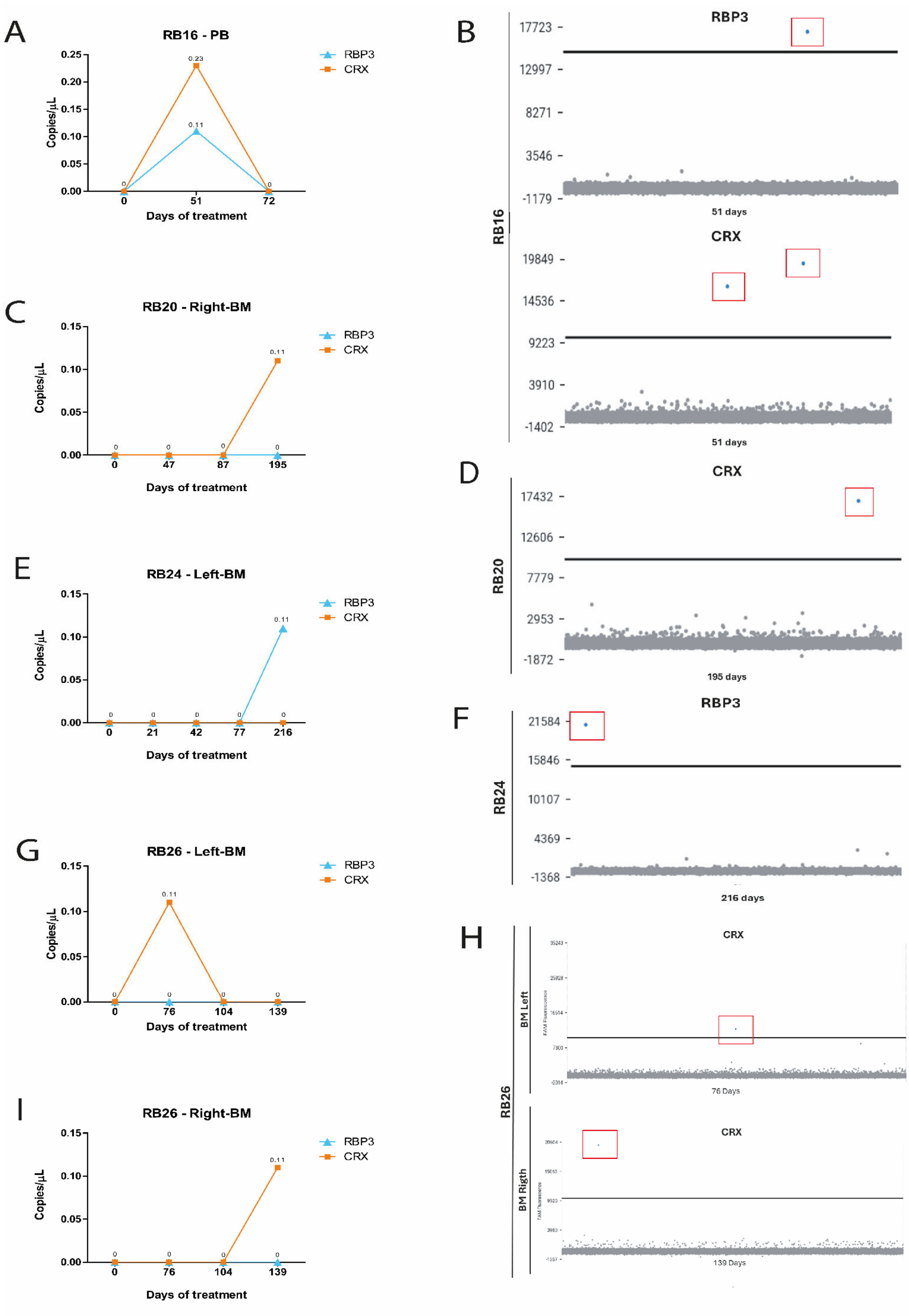

Supplementary Figure S4: Detection of *RBP3* and *CRX* gene expression in samples collected during treatment ins patients. Representative graphics and 1D scatter plots of patients RB16 (A and B), RB20 (C and D), RB24 (E and F), RB26 (G, H and I).
