# Supplementary material for: Digital PCR Quantification of a Circulating RBP3 and CRX RNA Signature Establishes a Liquid Biopsy Framework for Precision Monitoring of Retinoblastoma
Source: Int J Mol Sci. 2026 May 8;27(10):4177. doi: 10.3390/ijms27104177 (PMC13206994; doi:10.3390/ijms27104177)
Supplement: Supplementary file 1 [file ijms-27-04177-s001.zip › Supplementary Table S1.pdf]

Supplementary Table S1: Analysis of 433 samples and the overall comparison of digital PCR, myelogram and CSF cytology analysis;

Patient	Days of treatmet	Right-BM			Left-BM			CSF			PB	
		RBP3	CRX	Myelogram	RBP3	CRX	Myelogram	RBP3	CRX	CSF Cytology	RBP3	CRX
RB1	0	-	+	-	-	+	-	-	-	-	-	+
	481	+	+	-	+	+	-	-	-	-	-	-
	595	-	+	-	-	+	-				-	+
	TCTH 43				-	-	-				-	-
RB2	0	+	+	+	+	+	+	-	-	-	-	+
RB3	0	-	-	-	-	-	-	-	-	-	-	-
	48	-	-	-	-	-	-	-	-	-	-	-
RB4	0	-	-	-	-	-	-	-	-	-	-	-
RB5	0	-	-	-	-	-	-	-	-	-	-	-
RB6	0	-	-	-	-	+	-	-	-	-	-	-
RB7	0	+	+	+	+	+	+	+	+	+	+	+
	47	-	-	-	-	-	-	-	-	-	-	-
	TCTH 47	-	-	-	-	-	-	-	-	-	-	-
	TCTH 150	-	-	-	+	+	-	-	-	-	-	-
	TCTH 208	-	-	-	-	-	-	-	-	-	+	-
	TCTH 300	-	-	-	-	-	-	-	-	-	-	-
	TCTH 360	-	-	-	-	-	-	-	-	-	-	-
	TCTH 457	-	-	-	-	-	-	-	-	-	-	-
RB8	0	+	+	+	+	+	+	-	-	-	-	-
	102	-	-	-	+	-	-	-	-	-	-	-
	199	-	-	-	-	-	-	-	-	-	-	-
RB9	0	-	-	-	-	+	-	-	-	-	-	+
RB10	0	-	-	-	-	-	-	-	-	-	-	-
RB11	0	-	-	-	-	-	-	-	+	-	-	-

	23							+	-	-		
	44							-	-	-		
	115	-	-	-	-	-	-	-	-	-	-	-
	245	-	-	-	-	-	-	-	-	-	-	-
	329	-	-	-	+	-	-	-	-	-	-	-
	401	+	-	-	-	-	-	-	-	-	-	-
	490	-	-	-	+	-	-	-	-	-	-	-
**RB12**	0	-	-	-	-	+	-	-	-	-	-	-
**RB13**	0	+	+	+	+	+	+					
	49	-	-	-	-	-	-	-	-	+	-	-
	70	-	-	-	-	-	-	-	-	-	-	-
	91	+	-	-	+	-	-	-	-	-	-	-
	161	-	-	-	-	-	-	-	-	-	-	-
	219	-	-	-	-	-	-	-	-	-	-	-
	TCTH 30	-	-	-	-	-	-	-	-	-	-	-
	TCTH 90	-	-	-	-	-	-	-	-	-	-	-
**RB14**	0	-	-	-	-	-	-	-	-	-	-	-
	140	-	-	-	-	-	-	-	-	-	-	-
**RB15**	0	-	-	-	-	-	-	-	-	-	-	-
**RB16**	0	-	-	-	-	-	-	-	-	-	-	-
	51	-	-	-	-	-	-	-	-	-	+	+
	72	-	-	-	-	-	-	-	-	-	-	-
**RB17**	0	-	-	-	-	-	-	-	-	-	-	-
	42	-	-	-	-	-	-	-	-	-	-	-
	108	-	-	-	-	-	-	-	-	-	-	-
	178	-	-	-	-	-	-	-	-	-	-	-

**RB18**	0	-	-	-	-	-	-				-	-
	43	-	-	-	-	-	-				-	-
	64							-	-	+		
	83	-	-	-	-	-	-	-	-	+	-	-
**RB19**	0	-	-	-	-	-	-	-	-	-	-	-
**RB20**	0	-	-	-	-	-	-	-	-	-	-	-
	47	-	-	-	-	-	-	-	-	-	-	-
	87	-	-	-	-	-	-	-	-	-	-	-
	195	-	-	-	+	-	-	-	-	+	-	-
**RB21**	0	-	-	-	-	-	-	-	-	-	-	-
**RB22**	0	-	-	-	+	-	-	-	-	-	-	-
**RB23**	0	-	+	-	+	-	-	-	-	-	-	-
	153	-	-	-	-	-	-	-	-	-	-	-
**RB24**	0	-	-	-	-	-	-	-	-	-	-	-
	24	-	-	-	-	-	-	-	-	-	-	-
	42	-	-	-	-	-	-	-	-	-	-	-
	77	-	-	-	-	-	-	-	-	-	-	-
	216	-	-	-	-	+	-	-	-	-	-	-
**RB25**	0	+	+	+				-	-	-		
	45	-	-	-	-	-	-	-	-	-	-	+
	66	-	-	-	-	-	-					
	179	-	-	-	-	-	-	-	-	-	-	-
	240	-	+	-	-	-	-	-	-	-	-	-
**RB26**	0	-	-	-	-	-	-	-	-	-	-	-
	76	-	-	-	-	+	-	-	-	-	-	-
	104	-	-	-	-	-	-	-	-	-	-	-

	139	-	-	-	+	-	-	-	-	-	-	-
**RB27**	0	-	-	-	-	+	-	-	-	-	-	-
	96	-	-	-	-	-	-	-	-	-	-	-
**RB28**	0	+	+	+	+	+	+	-	-	-	+	+
	49	-	-	-	-	-	-	-	-	-	+	-
	118							-	-	+		
	188	-	-	-	-	-	-	-	-	+	-	-
	213							-	-	-		
**RB29**	0	-	-	-	-	-	-	-	-	-	-	-
**RB30**	0	-	-	-	-	-	-	-	-	-	-	-
**RB31**	0	-	-	-	-	-	-	-	-	-	-	-
**RB32**	0	+	+	-	-	+	-	-	-	-	+	-
	12	+	+	+	-	+	-	-	-	+	-	+
	61	-	-	-	-	-	-	+	-	+	-	-
**RB33**	0							-	-	+		
**RB34**	0	+	+	+				-	-	-	+	+
	21	-	+	-	-	-	-	-	-	-	-	-
	42	-	-	-	-	-	-	-	-	-	-	-
	78	-	-	-	-	-	-					
**RB35**	0	-	-	-	-	-	-	-	-	-	-	-
**RB36**	0	+	+	-	+	+	-	-	+	-	-	-
	68	-	-	-	-	-	-	-	+	-	-	-
	90	-	+	-	-	+	-	-	-	-	-	-
**RB37**	0	-	-	-	-	-	-	-	-	-	-	-
**RB38**	0	-	-	-	-	+	-	-	-	-	-	-
	119	-	+	-	-	-	-	-	-	-	-	-

**RB39**	0	-	-	-	-	-	-	-	+	-	-	-
**RB40**	0	-	-	-	-	-	-	+	-	-	-	-
**RB41**	0	-	-	-	-	-	-	-	-	-	-	-
**RB42**	0	-	-	-	-	-	-	-	-	-	-	-
	0	+	-	-	-	-	-	-	-	+	-	-
**RB43**	46	+	+	-	-	+	-	-	-	-	-	-
	74	-	-	-	-	+	-	-	-	-	-	-
**RB44**	0	+	-	-	-	-	-	-	-	-	-	-
**RB45**	0	-	-	-	-	-	-	-	-	-	-	-
**RB46**	0	-	-	-	+	-	-	-	+	-	-	-
**RB47**	0	-	-	-	-	+	-	-	-	-	-	-
**RB48**	0	-	-	-	+	-	-	-	-	-	-	-
**RB49**	0	+	+	+	+	+	+	-	+	-	+	+
**RB50**	0	-	-	-	-	-	-	-	-	-	-	-

Right-BM: Right bone marrow; Left-BM: Left bone marrow; CSF: cerebrospinal fluid; PB: peripheral blood.
