# Supplementary material for: Digital PCR Quantification of a Circulating RBP3 and CRX RNA Signature Establishes a Liquid Biopsy Framework for Precision Monitoring of Retinoblastoma
Source: Int J Mol Sci. 2026 May 8;27(10):4177. doi: 10.3390/ijms27104177 (PMC13206994; doi:10.3390/ijms27104177)
Supplement: Supplementary file 1 [file ijms-27-04177-s001.zip › Supplementary Table S2.pdf]

Supplementary Table S2: Positive samples for digital PCR assay and myelogram and CSF cytology analysis in 433 samples;

Neoplastic cell detection	Myelogram method						Neoplastic cell search		
	Right-BM (n° of samples)			Left-BM (n° of samples)			CSF (n° of samples)		
	Positive	Negative	Total	Positive	Negative	Total	Positive	Negative	Total
**dPCR method**									
**Positive**	9	15	24	6	27	33	2	8	10
**Negative**	0	85	85	0	75	75	11	89	100
**Total**	9	100	109	6	102	108	13	97	110

Right-BM: Right bone marrow; Left-BM: Left bone marrow; CSF: cerebrospinal fluid.
