# Supplementary material for: Digital PCR Quantification of a Circulating RBP3 and CRX RNA Signature Establishes a Liquid Biopsy Framework for Precision Monitoring of Retinoblastoma
Source: Int J Mol Sci. 2026 May 8;27(10):4177. doi: 10.3390/ijms27104177 (PMC13206994; doi:10.3390/ijms27104177)
Supplement: Supplementary file 1 [file ijms-27-04177-s001.zip › Supplementary Table S3.pdf]

Supplementary Table S3: Follow-up of 22 patients during the treatment.

Patient ID	Gender	Laterality	Tumor	Follow-up number	Positive for genes
RB1	M	B	E	4	Yes
RB3	M	B	E	2	No
RB7	M	U	E	8	Yes
RB8	M	B	E	3	Yes
RB11	M	U	E	7	Yes
RB13	F	U	E	8	Yes
RB14	M	U	I	2	No
RB16	F	T	E	3	Yes
RB17	F	T	E	4	No
RB18	F	B	I	4	No
RB20	M	U	E	4	Yes
RB23	M	B	I	2	Yes
RB24	M	U	I	5	No
RB25	F	B	I	5	Yes
RB26	F	B	E	4	Yes
RB27	F	B	I	2	Yes
RB28	M	U	E	5	Yes
RB32	F	U	E	3	Yes
RB34	F	U	E	4	Yes
RB36	F	U	I	3	Yes
RB38	M	U	I	2	Yes
RB43	F	T	I	3	Yes
